# Performance Enhancement of Hole Transport Layer-Free Carbon-Based CsPbIBr_2_ Solar Cells through the Application of Perovskite Quantum Dots

**DOI:** 10.3390/nano14201651

**Published:** 2024-10-14

**Authors:** Qi Yu, Wentian Sun, Shu Tang

**Affiliations:** 1 Huailai Shengshi New Energy Technology Co., Ltd., Zhangjiakou 075400, China; 13811236212@139.com; 2 School of Science, China University of Geosciences Beijing, Beijing 100083, China; w1063783539@163.com

**Keywords:** quantum dots, anti-solvent additive, interface, carbon-based CsPbIBr_2_ PSCs, long-term stability

## Abstract

CsPbIBr_2_, with its suitable bandgap, shows great potential as the top cell in tandem solar cells. Nonetheless, its further development is hindered by a high defect density, severe carrier recombination, and poor stability. In this study, CsPbI_1.5_Br_1.5_ quantum dots were utilized as an additive in the ethyl acetate anti-solvent, while a layer of CsPbBr_3_ QDs was introduced between the ETL and the CsPbIBr_2_ light-harvester film. The combined effect of these two QDs enhanced the nucleation, crystallization, and growth of CsPbIBr_2_ perovskite, yielding high-quality films characterized by an enlarged crystal size, reduced grain boundaries, and smooth surfaces. And a wider absorption range and better energy band alignment were achieved owing to the nano-size effect of QDs. These improvements led to a decreased defect density and the suppression of non-radiative recombination. Additionally, the presence of long-chain organic molecules in the QD solution promoted the formation of a hydrophobic surface, significantly enhancing the long-term stability of CsPbIBr_2_ PSCs. Consequently, the devices achieved a PCE of 9.20% and maintained an initial efficiency of 85% after 60 days of storage in air. Thus, this strategy proves to be an effective approach for the preparation of efficient and stable CsPbIBr_2_ PSCs.

## 1. Introduction

Organic–inorganic hybrid perovskite films have been widely used in the field of photovoltaics due to their favorable bandgap and high light absorption coefficient [1,2,3,4]. However, the presence of A-site organic cations (such as FA^+^ ions and MA^+^ ions) makes them chemically unstable under conditions such as water vapor and high temperature [5,6,7,8]. In addition, ion migration under illumination also limits the photostability of this material [9,10], thus limiting its further development. The photo and thermal stability of all inorganic CsPbI_3_ perovskite films is much better than that of organic–inorganic hybrid perovskite films, but the phase stability is poor, and it is easy to transform from the α-black cubic phase to the non-perovskite β-yellow phase at room temperature [11,12,13]. The stability of CsPbBr_3_ is excellent, but its bandgap of 2.3 eV results in a narrow light-absorbing range, rendering it unsuitable for use as a light-absorbing layer in perovskite solar cells (PSCs) [14,15]. In light of these considerations, PSCs comprising CsPbIBr_2_ thin films as the light-absorbing layer have garnered considerable attention, given their potential to balance device efficiency and stability [16,17]. Compared to CsPbBr_3_, CsPbIBr_2_ exhibits a narrower forbidden bandwidth of approximately 2.05 eV and displays a significantly enhanced thermal stability (>460 °C) relative to CsPbI_3_.

At present, the CsPbIBr_2_ PSCs reported in the literature mostly adopt a device structure of TCO glass/electron transport layer (ETL)/perovskite/hole transport layer (HTL)/metal electrode [18,19,20]. The metal electrode mostly uses noble metals such as Au [17] and Ag, and the hole transport layer mostly uses expensive and unstable organic materials such as Spiro-MeOTAD [19], which is not conducive to industrial development. The adoption of a hole transport layer-free (HTL-free) structure in carbon electrode PSCs, which entails the elimination of traditional metal electrodes and hole transport materials, has the potential to significantly reduce the preparation cost, thereby enhancing cost-effectiveness and sustainability. Nevertheless, the power conversion efficiency (PCE) of carbon-based CsPbIBr_2_ PSCs without a hole transport layer remains relatively low. In 2023, Zhao et al. enhanced the efficiency of carbon-based CsPbIBr_2_ solar cells from 6.63% to 8.09% by introducing lithium doping into the electron transport layer [21]. In 2024, Lan et al. enhanced the efficiency of carbon-based CsPbIB_2_ solar cells from 8.26% to 10.23% by modifying the interface between the perovskite and carbon electrode with 2-iodoaniline [22]. Zhang et al. applied (3-bromopropyl) trimethylammonium bromide as an interface regulator to reduce energy offset and facilitate hole extraction and delocalization, improving photosensitive layer quality and inhibiting carrier recombination, and finally achieved a PCE of 11.26% [23].

Quantum dots (QDs) offer several advantageous features, including the beneficial nano-size effect, unique multi-exciton generation, superior phase stability, and remarkable hysteresis suppression [24,25]. The nano-size effect, arising from quantum confinement at the nanometer scale of QDs, facilitates tunable bandgap properties. This characteristic enables the efficient absorption of visible and infrared (IR) light, and enhances the transport of charges within the material [26,27]. Modifying PSCs with perovskite QDs is a promising strategy to maintain the merits of both perovskite and QDs. In recent years, low-dimensional QDs have exhibited significant potential as additives in anti-solvents and interface treatment materials, offering a number of advantageous properties, including an easily regulated bandgap, high stability, and low exciton energy [28]. In 2020, Xu et al. introduced a novel method for nucleation assistance by utilizing CsPbBr_3_ QDs as additives in ethyl ether anti-solvent, facilitating the preparation of high-quality, smooth, pinhole-free MAPbI_3_ films [29]. This innovative approach resulted in an efficiency improvement to 20.17%, and after aging for 1000 h at 50% relative humidity, the initial efficiency was retained at 85%. Khorshidi synthesized functionalized graphene quantum dots (AGQDs) featuring carbonyl, amine, and long hydrophobic alkyl chains as effective additives in a mixture of toluene and hexane anti-solvents, resulting in a MAPbI_3_:AGQDs gradient heterojunction structure. This composition produced a hydrophobic, uniform, and dense perovskite film, where the interaction between AGQDs and non-coordinated Pb^2+^ significantly passivated the surface defects of the perovskite. The champion device achieved an efficiency increase from 16.00% to 19.10%, coupled with excellent stability [30]. Furthermore, Yang et al. reported that carbon QDs, prepared by pulsed laser irradiation, added in chlorobenzene anti-solvent, could effectively passivate defects at the grain boundaries of MAPbI_3_. Compared to pure chlorobenzene anti-solvent, the efficiency of carbon-based HTL-free PSCs improved from 13.11% to 14.95% [31]. Additionally, Qi et al. introduced PbS QDs between SnO_2_ and CsPbIBr_2_, and improved the efficiency of the device from 7.00% to 9.09% by reducing interface defects [32]. In 2023, Liu et al. introduced CsPbIBr_2_ QDs prepared by the thermal injection method as an interface layer into CsPbIBr_2_/carbon, increasing the efficiency of PSCs from 6.67% to 7.36% [33]. Thus, perovskite QDs were demonstrated to be performance-optimization materials for PSCs due to their similar structure to the absorption layer and adjustable bandgap.

In this study, we present a PSC comprising the FTO/TiO_2_/CsPbBr_3_ QD/CsPbIBr_2_/carbon configuration. Initially, the CsPbIBr_2_ light-absorption layer was prepared by a one-step anti-solvent method where the CsPbI_1.5_Br_1.5_ QDs were employed as an additive in the ethyl acetate (EA) anti-solvent. To our knowledge, CsPbIBr_2_ is susceptible to defects such as pinholes and Pb vacancies during the preparation process, which can act as recombination centers for charge carriers, consequently diminishing the device efficiency [34,35]. To address this issue, an enhanced solvothermal method was employed to synthesize CsPbI_1.5_Br_1.5_ QDs, which were then introduced into EA as an additive to regulate the crystallization kinetics of the CsPbIBr_2_ thin film. This approach aimed at improving the crystalline quality of the CsPbIBr_2_ thin film, mitigating Pb vacancies, and thereby enhancing the photovoltaic performance of the device. The innovative strategy of utilizing QD solutions as anti-solvents to manipulate the crystallization kinetics of all-inorganic perovskite film, leading to the enhanced efficiency of carbon-based HTL-free devices, still lacks comprehensive analysis. Subsequently, an impressive PCE of 7.96% was achieved, signifying a substantial enhancement compared to the 5.48% attained by using pure EA anti-solvent treatment.

Based on this, we subsequently served CsPbBr_3_ QDs as an interface modification layer between the ETL and perovskite layer. On one hand, CsPbBr_3_ can act as “seeds” for the growth of CsPbIBr_2_ perovskite, regulating its nucleation [36]. On the other hand, after the QD solvent of cyclohexane evaporation, a porous structure is formed on the surface of TiO_2_, providing space for the growth of CsPbIBr_2_ perovskite and promoting the increase in grain size. The interface modification of TiO_2_/CsPbIBr_2_ by CsPbBr_3_ QDs not only improves the crystalline quality of perovskite films, but also passivates interface defects, promotes carrier transport, and enhances device efficiency. In addition, the QDs used in this work were obtained by an improved solvothermal method, which has the advantages of simple processing, low reaction temperature, and easy observation compared to traditional thermal injections and ligand-assisted reprecipitation methods. Following the optimization of the two kinds of QDs, a gradient energy band arrangement was built and the PCE of the HTL-free carbon-based PSCs eventually reached 9.20%, demonstrating a significant improvement in comparison to the unmodified device.

## 2. Materials and Methods

### 2.1. Materials

All chemicals were used without further treatment or purification. Titanium (IV) isopropoxide (99.999%), oleic acid (OA, 90%), oleylamine (OAm, 70%), 1-octadecene (ODE, 90%), ethyl acetate (EA, 99%), cyclohexane (90%), and Cs_2_CO_3_ (99%) were purchased from Aladdin Corporation (Shanghai, China). PbBr_2_ (99.99%) and CsI (99.9%) were purchased from Xi’an Yuri Solar Co., Ltd. (Xi’an, China), and dimethyl sulfoxide (DMSO, 99.7%) was purchased from Macklin reagent. Shanghai Mater Win New Materials Co., Ltd. (Shanghai, China) provided the conductive carbon paste.

### 2.2. Synthesis of CsPbI_1.5_Br_1.5_ and CsPbBr_3_ QDs

**Figure 1:**
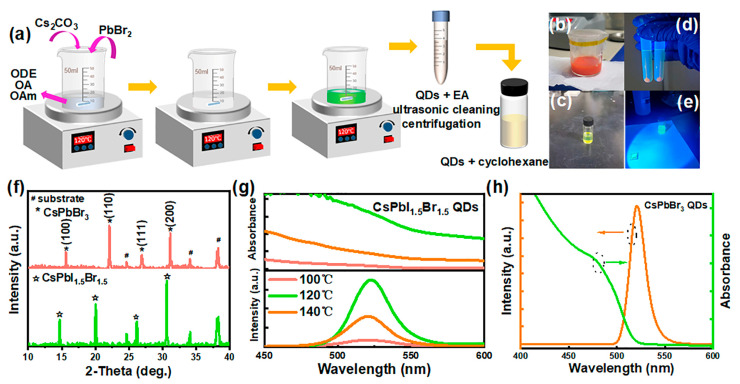
Schematic representation of QDs prepared by the solvothermal method (**a**); images of CsPbI_1.5_Br_1.5_ QDs and CsPbBr_3_ QDs in sunlight (**b**,**c**), and UV light (**d**,**e**); XRD patterns of CsPbI_1.5_Br_1.5_ (green) and CsPbBr_3_ (orange) QDs (**f**); UV-vis absorption (upper) and PL spectra (lower) of CsPbI_1.5_Br_1.5_ QDs (**g**) and UV-vis absorption (green) and PL spectra (orange) of CsPbBr_3_ QDs (**h**).

Using an optimized solvothermal approach, we successfully synthesized CsPbI_1.5_Br_1.5_ and CsPbBr_3_ QDs following the process presented in Figure 1a. 

#### 2.2.1. Preparation of CsPbI_1.5_Br_1.5_ QDs

The synthesis of CsPbI_1.5_Br_1.5_ QDs employing an enhanced solvothermal approach begins with the combination of 0.1 mmol Cs_2_CO_3_, 0.15 mmol PbBr_2_, and 0.15 mmol PbI_2_ in a mixed solvent of 10 mL of ODE, 1.5 mL OA, and Oam ligands, which were stirred at 100, 120, and 140 °C until dissolved, respectively. And then, the critical observation of the transition from opaque white to colorless followed by the rapid manifestation of a vibrant orange signifies the completion of the reaction. The termination of the heating process precisely 30 s post-color change and centrifugation yielded the desired CsPbI_1.5_Br_1.5_ QD samples.

#### 2.2.2. Preparation of CsPbBr_3_ QDs

Similarly, 0.1 mmol of Cs_2_CO_3_ and 0.3 mmol of PbBr_2_ were added into of a 10mL ODE, 1.5 mL OA, and 1.5mL Oam mixture, thoroughly stirred at 120 °C until the color of the solution abruptly changed from a cloudy white to colorless, followed by a rapid transition to a vivid green. After observing this color change for 30 s, heating was discontinued and centrifugation was applied to yield the desired CsPbBr_3_ QDs.

The two types of QD precipitates were redispersed in 4 mL EA, respectively, for further purification of ultrasonic cleaning for 5 min and then centrifuging, and the desired two kinds of QDs were obtained. The photos of the synthesized products showcase their appearance under sunlight and ultraviolet (UV) light. The CsPbI_1.5_Br_1.5_ quantum dots display a prominent reddish-orange (Figure 1b) when exposed to sunlight, while their luminescence is evidently enhanced under UV light (Figure 1d). In the next section, the cyclohexane solutions with different QD concentrations were prepared according to the specific requirement.

### 2.3. Device Fabrication

The FTO conductive glass, composed of F-doped tin oxide, underwent an initial cleaning with a dedicated conductive glass cleaning solution, followed by sequential sonication in ethanol, isopropanol, and deionized water for 15 min each. This cleaning process was subsequently complemented by a 5 min treatment in a plasma cleaner set to 20 W to enhance surface wettability.

Following the surface preparation, a TiO_2_ compact layer solution—comprising isopropyl titanate and hydrochloric acid dissolved in anhydrous ethanol—was deposited onto the FTO substrate by spin-coating at 3000 rpm for 30 s. This TiO_2_ layer was then thermally annealed on a hotplate at 150 °C for 15 min, followed by further annealing in a muffle furnace at 500 °C for an additional 30 min. Subsequently, the sample was immersed in a TiO_2_ aqueous solution and subjected at 70 °C for 30 min. After drying the sample with a hair dryer, it was once again placed in the muffle furnace for annealing treatment at 500 °C for 30 min, resulting in the formation of the FTO/c-TiO_2_ sample.

In this work, we proposed two optimized approaches for the CsPbIBr_2_ PSCs. The first approach entailed the construction of PSCs following the architecture of FTO/TiO_2_/CsPbIBr_2_/carbon. Initially, a cyclohexane solution containing CsPbI_1.5_Br_1.5_ QDs was prepared at a concentration of 4.25 mg/mL, which was then combined with EA in volume ratios of 0%, 20%, 25%, and 30% to formulate QD anti-solvents. The perovskite precursor solution, consisting of 1 M CsI and PbBr_2_ dissolved in DMSO, was subsequently spin-coated onto the TiO_2_ substrate applying a two-step program of 1500 rpm for 20 s and 5000 rpm for 60 s. Then, 200 μL of the as-prepared QD anti-solvent was dripped in the last 30 s and the sample was annealed at 200 °C for 15 min to facilitate the formation of the desired CsPbIBr_2_ light-absorbing layer.

The second optimization method focused on the fabrication of PSCs structured as FTO/TiO_2_/CsPbBr_3_ QD/CsPbIBr_2_/carbon. Utilizing the results from the above experiments, the optimal concentration of CsPbI_1.5_Br_1.5_ QDs in the anti-solvent was determined. A layer of CsPbBr_3_ QDs was then introduced between the ETL and CsPbIBr_2_ for interface modification. Following this, cyclohexane solutions of CsPbBr_3_ QDs were prepared at varying concentrations of 0, 0.2, 0.4, and 0.6 mg/mL, and deposited onto the TiO_2_ layer at a spinning speed of 4000 rpm for 30 s. After applying the prior deposition method for the CsPbIBr_2_ layer, a carbon paste was finally doctor-bladed, followed by annealing at 120 °C for 15 min.

## 3. Results

### 3.1. Charecterization of CsPbI_1.5_Br_1.5_ and CsPbBr_3_ QDs

To verify the phase of the as-obtained two kinds of QDs, the X-ray diffraction (XRD) patterns were conducted by spinning the QD solution onto the FTO substrate (Figure 1f). For the CsPbI_1.5_Br_1.5_ QDs, the diffraction peaks at 14.6°, 20.8°, and 29.7° correspond to (100), (110), and (200) planes of CsPbI_1.5_Br_1.5_ [37]. In terms of CsPbBr_3_ QDs, the peaks at 15.6°, 22.0°, and 31.1° can be assigned to the (100), (110), and (200) planes of CsPbBr_3_ [38]. A slight shift of the crystal planes to higher angles can be observed with the bromine content increasing, owing to a decrease in the lattice constant due to the smaller ionic radius of Br^−^ compared to I^−^. The photoluminescent (PL) peaks of the CsPbI_1.5_Br_1.5_ perovskite QDs, fabricated at various temperatures, were concentrated around 580 nm, indicating pronounced emission characteristics (Figure 1g). At a heating temperature of 100 °C, the PL intensity was relatively weak; furthermore, upon increasing the temperature to 120 °C, there was a notable enhancement in PL intensity, signifying a reduction in non-radiative recombination processes associated with defects. Conversely, when the temperature was further elevated to 140 °C, there was a decline in PL intensity, suggesting that excessively high temperatures adversely affect the crystallization quality of the perovskite QDs. The ultraviolet-visible (UV-vis) spectra of the CsPbI_1.5_Br_1.5_ QDs at varying heating temperatures also highlighted the best light absorption capability observed at 120 °C (Figure 1g). In addition, the CsPbBr_3_ QDs were fabricated at 120 °C, exhibiting a bright-yellow emission under sunlight (Figure 1c) and vibrant green fluorescence when subjected to UV light (Figure 1e). The UV-vis absorption and PL spectra (Figure 1h) suggested that the PL peak for the CsPbBr_3_ QDs was located at approximately 520 nm.

### 3.2. Characterization of FTO/TiO_2_/CsPbIBr_2_/Carbon-Structured PSCs

#### 3.2.1. Morphological Properties

**Figure 2:**
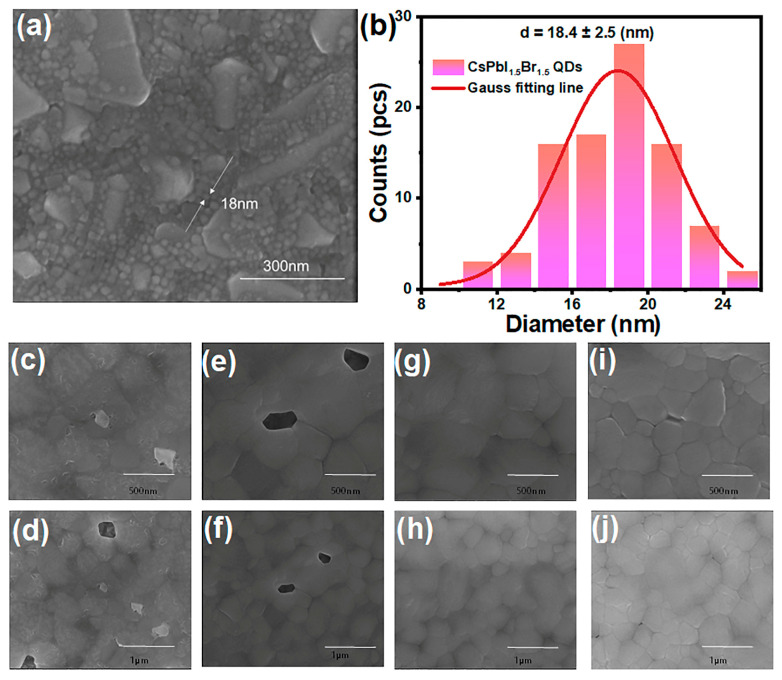
SEM image (**a**) and particle size statistics (**b**) of CsPbI_1.5_Br_1.5_ QDs; SEM images of 0% (**c**,**d**), 20% (**e**,**f**), 25% (**g**,**h**), and 30% (**i**,**j**) CsPbI_1.5_Br_1.5_ QD additive in EA.

To study the surface morphology of CsPbI_1.5_Br_1.5_ perovskite QDs, a solution of the as-prepared QDs in cyclohexane was spin-coated onto an FTO substrate, followed by characterization via scanning electron microscopy (SEM) (Figure 2a). The resulting QDs demonstrated a consistent size distribution. The particle size distribution indicates that the average dimension of the synthesized QDs was approximately 18.4 nm (Figure 2b). Subsequently, to investigate the effect of CsPbI_1.5_Br_1.5_ QDs as an additive in EA anti-solvent on the properties of CsPbIBr_2_ perovskite films, we took varying volume ratios of 0%, 20%, 25%, and 30% of a 4.25 mg/mL cyclohexane solution of the QDs into EA anti-solvents, and then prepared the CsPbIBr_2_ perovskite films employing a one-step anti-solvent method. Notably, in the absence of QDs, the films presented big voids, a rough surface, and low coverage (Figure 2c,d). Additionally, the small crystallite sizes and high density of grain boundaries significantly contributed to the increased defect state density in the perovskite films, resulting in severe non-radiative recombination and leakage currents detrimental to charge carrier transport, ultimately leading to decreased device efficiency. Upon introducing 20% CsPbI_1.5_Br_1.5_ QDs into the anti-solvent, substantial improvements in crystallite dimensions in the surface morphology of the CsPbIBr_2_ films were observed (Figure 2e,f). Increasing the QD concentration to 25% further refined the surface morphology, yielding a smooth and dense film with no visible pinholes and significantly larger grains accompanied by fewer boundaries (Figure 2g,h). This enhancement can be attributed to the function of CsPbI_1.5_Br_1.5_ QDs as nucleation sites during the crystallization of CsPbIBr_2_, where an optimal concentration facilitates nucleation, crystallization, and growth, resulting in larger grains and a more uniform surface. Additionally, Pb^2+^ ions from the QDs may occupy Pb vacancies within the CsPbIBr_2_ films, further enhancing the crystalline quality. However, when the QD concentration was increased to 30%, pinholes appeared on the films (Figure 2i,j), revealing a decline in crystallization quality. This deterioration was attributed to the excessive number of nucleation sites resulting from the surplus of CsPbI_1.5_Br_1.5_ QDs in the wet CsPbIBr_2_ films, which inhibited CsPbIBr_2_ crystal growth and consequently produced smaller grain boundaries and rougher surfaces. Alongside that, excessive organic ligands such as OA and Oam accumulating on the surface of the CsPbIBr_2_ films also negatively affected the crystallization quality. Except morphological modification induced by QDs observed in this way, there are other methods for morphology improvement at the nano-scale in perovskite films, such as the application of metallic nanoparticles [39].

#### 3.2.2. Photovoltaic Properties

The space charge-limited current (SCLC) characteristics [40] of FTO/TiO_2_/CsPbIBr_2_/carbon-structured PSCs derived from anti-solvents without QDs compared to those with 25% CsPbI_1.5_Br_1.5_ QDs were conducted (Figure S1a). Notably, the trap filled limit voltage (V_TFL_) of the fabricated devices decreased from an unoptimized value of 0.92 V to 0.71 V. This reduction in V_TFL_ indicates that the modified perovskite layer exhibited a lower defect state density. Additionally, the dark J–V curves for the mentioned two devices revealed a significant decline in leakage current from 10^−7^ mA/cm^2^ to 10^−9^ mA/cm^2^ when adding 25% CsPbI_1.5_Br_1.5_ QDs into anti-solvent, further substantiating the reduction in defects within the CsPbIBr_2_ light-absorbing layer (Figure S1b).

**Figure 3:**
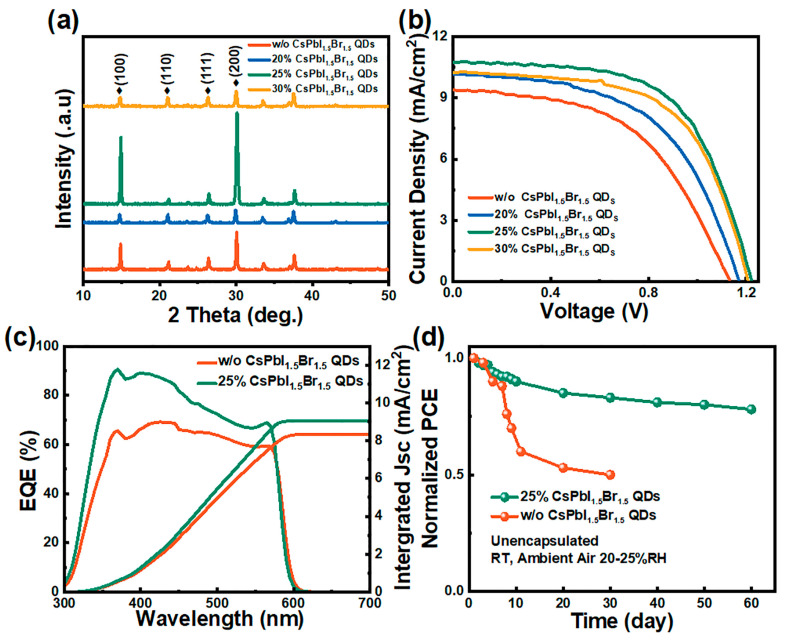
XRD patterns (**a**) and the best-performing devices’ J–V curves (**b**) after varying CsPbI_1.5_Br_1.5_ QD additive amount modification; the EQE and integrated Jsc curves (**c**) and stability tests (**d**) of the unoptimized and the 25%-treated devices.

The XRD patterns of CsPbIBr_2_ films were obtained using EA with varying proportions of CsPbI_1.5_Br_1.5_ QD additives (Figure 3a), of which the peaks at 14.8°, 21.1°, 26.3°, and 30.1° were assigned to the (100), (110), (111), and (200) planes of CsPbIBr_2_ [33]. As the ratios of CsPbI_1.5_Br_1.5_ QDs in the anti-solvent increased from 0% to 25%, significant enhancements in the diffraction intensities of the dominant (100) and (200) planes were noticed [40], while with further increasing the ratio to 30%, the intensity decreased evidently, indicating that the addition of an appropriate amount of CsPbI_1.5_Br_1.5_ QDs improved the crystalline of the CsPbIBr_2_ perovskite, which was consistent with the SEM images (Figure 2). The J–V curves of carbon-based HTL-free CsPbIBr_2_ PSCs prepared with different ratios of CsPbI_1.5_Br_1.5_ QDs in the anti-solvent were recorded (Figure 3b), with detailed parameters such as open-circuit voltage (Voc), short-circuit current (Jsc), fill factor (FF), and power conversion efficiency (PCE) summarized in Table 1. The CsPbIBr_2_ PSCs using pure EA delivered a low PCE of 5.48%, along with a Voc of 1.12 V, a Jsc of 9.39 mA/cm^2^, and a FF of 52.11%, while the device based on 20% CsPbI_1.5_Br_1.5_ QD treatment showed an enhanced PCE of 7.42%, coupled with a Voc of 1.20 V, a Jsc of 10.25 mA/cm^2^, and a FF of 60.32%. A higher PCE of 7.96% was obtained using 25% CsPbI_1.5_Br_1.5_ QD additive in the EA, with the value of Voc, Jsc, and FF improved to 1.21 V, 10.73 mA/cm^2^, and 61.31%, respectively, while with further increasing the ratio of CsPbI_1.5_Br_1.5_ QDs to 30%, the PCE decreased to 6.45%, along with a Voc of 1.17 V, a Jsc of 10.15 mA/cm^2^, and a FF of 54.31%. The best PCE of 7.96% was achieved in 25% QD additive device, compared to the 5.48% of the control one, showing a remarkable enhancement of 55%. This improvement was attributed to the enhanced crystallization quality of the light-absorbing layer and a reduction in defect states density. The external quantum efficiency (EQE) curves (Figure 3c) for CsPbIBr_2_ PSCs indicated that in the PSCs incorporating 25% CsPbI_1.5_Br_1.5_ QD additives in anti-solvent, the integrated current raised from 8.35 mA/cm^2^ to 9.05 mA/cm^2^, consistent with the increase in Jsc observed in the J–V curves (Figure 3b). This further highlighted the pronounced effect of using QDs as an anti-solvent additive for film modification. To evaluate the stability of the as-prepared carbon-based HTL-free CsPbIBr_2_ PSCs, stability tests were conducted (Figure 3d). The PSC optimized with 25% QDs in the anti-solvent maintained approximately 80% of their initial PCE after 60 days, whereas the control PSC without optimization only retained 80% PCE after 10 days. Over time, the PCE of the unoptimized device declined sharply, dropping to about 50% of the initial value after 30 days. This observation suggested that the long-chain organic ligands located in the QD solution can coordinate with the grain boundaries, forming a hydrophobic surface that stabilized the perovskite structure, thereby increasing long-term stability. Furthermore, we conducted statistics on the photovoltaic parameters of eight devices (Figure S2) and summarized the results (Table S1). Obviously, CsPbI_1.5_Br_1.5_ QDs acted as additives in EA, significantly improving the optoelectronic properties of the devices, and the statistical data of PSCs were indicative of the good reproducibility of this method.

### 3.3. Characterization of FTO/TiO_2_/CsPbBr_3_ QD/CsPbIBr_2_/Carbon-Structured PSCs

#### 3.3.1. Morphological Properties

Based on the above results of the carbon-based HTL-free CsPbIBr_2_ PSCs, we applied an interfacial modification of inserting a CsPbBr_3_ QD layer between the ETL TiO_2_ and CsPbIBr_2_ film to further improve the photovoltaic performances of the PSCs.

**Figure 4:**
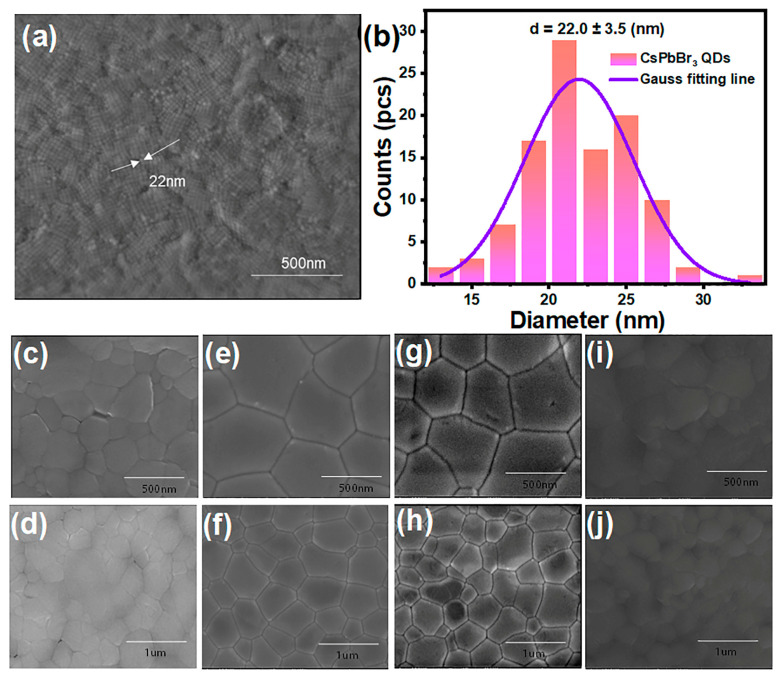
SEM image (**a**) and particle size distribution (**b**) of CsPbBr_3_ QDs; SEM images of 0 (**c**,**d**), 0.2 (**e**,**f**), 0.4 (**g**,**h**), and 0.6 mg/mL (**i**,**j**) CsPBr_3_ QD layers on the TiO_2_ films.

The surface morphology analysis of CsPbBr_3_ QDs on the FTO substrate, was characterized by SEM image (Figure 4a), displaying consistent dimensions and regular shape features. Additionally, the size distribution plot of CsPbBr_3_ QDs indicated an average dimension of approximately 22.0 nm (Figure 4b). To investigate the effect of the CsPbBr_3_ QD layer on the CsPbIBr_2_ films, we adjusted the CsPbBr_3_ QD spin-coating concentration to 0, 0.2, 0.4, and 0.6 mg/mL, respectively, and then deposited the CsPbIBr_2_ film according to the method aforementioned in the experimental section. According to their SEM images, the absence of the CsPbBr_3_ QD layer on the ETL resulted in diminished grain size and non-uniform surface morphology of the perovskite film (Figure 4c,d). In contrast, the application of 0.2 mg/mL QD solution substantially enlarged the grain size and improved the surface smoothness of the perovskite film (Figure 4e,f). Optimal film quality was achieved with the concentration of 0.4 mg/mL QDs (Figure 4g,h). However, further increasing the concentration of 0.6 mg/mL CsPbBr_3_ QDs led to a deterioration in film quality, attributable to an excess of organic ligands blocking the electron transmission (Figure 4i,j). Consequently, the SEM findings suggested that the perovskite layer spun onto a 0.4 mg/mL concentration of the CsPbBr_3_ QD layer exhibited superior surface morphology and grain size. The XRD analysis from the FTO/TiO_2_/CsPbBr_3_ QD/CsPbIBr_2_ structure indicated an upsurge in diffraction peak intensity at the (100) and (200) planes post-quantum dot layer modification, notably pronounced with the use of 0.4 mg/mL of CsPbBr_3_ QDs on the ETL (Figure S3). This enhancement underscored the superior diffraction peak intensity, signifying the enhancement in perovskite film quality following interfacial modification.

#### 3.3.2. Photovoltaic Properties

**Figure 5:**
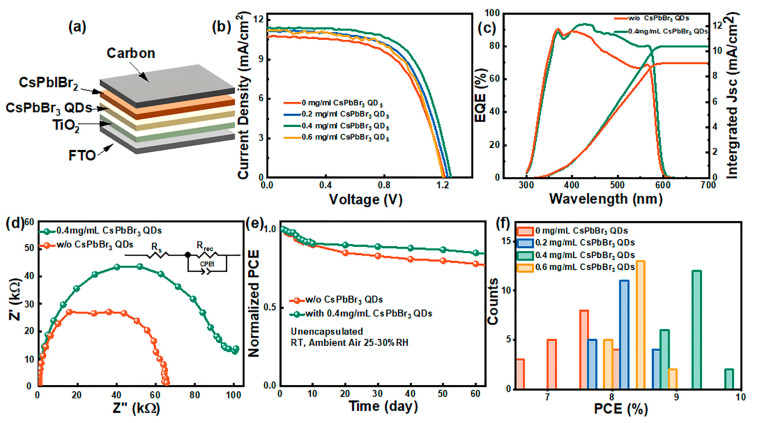
CsPbIBr_2_ PSC configuration (**a**); J–V curves of devices modified by different concentrations of CsPbBr_3_ QDs (**b**); the EQE curves (**c**), Nyquist plots, inset: equivalent circuit diagram (**d**), stability tests (**e**) of devices optimized with 0 and 0.4 mg/mL CsPbBr_3_ QD layers and the reproducibility of the devices (**f**).

To investigate the impact of the CsPbBr_3_ QD layer on the photovoltaic performances of the devices with a structure of FTO/TiO_2_/CsPbBr_3_ QD/CsPbIBr_2_/carbon (Figure 5a), it can be concluded from the J–V curves (Figure 5b) that depositing CsPbBr_3_ QDs with different concentrations on the TiO_2_ compact layer leads to improved device efficiency.

The corresponding photovoltaic parameters are provided in Table 2. The CsPbIBr_2_ PSCs without CsPbBr_3_ QDs delivered a low PCE of 7.96%, along with a Voc of 1.21 V, a Jsc of 10.73 mA/cm^2^, and a FF of 61.31%. In terms of the device based on 0.2 mg/mL CsPbBr_3_ QDs, it showed an enhanced PCE of 8.51%, coupled with a Voc of 1.23 V, a Jsc of 11.16 mA/cm^2^, and a FF of 61.99%. A PCE of 9.20% was obtained using a 0.4 mg/mL CsPbBr_3_ QD layer on the ETL, with the value of Voc, Jsc, and FF improved to 1.24 V, 11.41 mA/cm^2^, and 65.02%, respectively, while with further increasing the concentration of CsPbBr_3_ QDs to 0.6 mg/mL, the PCE decreased to 8.28%, along with Voc to 1.20 V, Jsc to 11.20 mA/cm^2^, and FF to 61.61%. The EQE curves of PSCs with and without CsPbBr_3_ QD layer modification on the compact layer were recorded (Figure 5c). The devices prepared with the concentration of 0.4 mg/mL of CsPbBr_3_ QDs onto the compact layer exhibited an integrated current value of 10.21 mA/cm^2^, showing a significant improvement compared to devices without this strategy. The dark J–V tests of CsPbIBr_2_ PSCs showed that the leakage current decreased from 10^−7^ mA/cm^2^ to 10^−8^ mA/cm^2^ when employing the CsPbBr_3_ QD layer (Figure S4a), indicating improved charge separation capability. It can be figured out that the V_TFL_ decreased from 0.95 V to 0.54 V, which revealed a reduction in trap density with the CsPbBr_3_ QD interfacial modification, suggesting reduced non-radiative recombination, leading to enhanced device performances and a lower defect density [38,40] (Figure S4b).

The electrochemical impedance spectroscopy (EIS) tests of devices were measured at a 0.5 V bias voltage. The Nyquist plot and equivalent circuit diagram indicated a decrease in series resistance (Rs) and an increase in charge recombination resistance (Rrec), reflecting improved charge transfer and reduced non-radiative recombination between electron transport and the light-absorption layers, benefiting from the nano-size of QDs (Figure 5d) [41]. Stability tests showed that devices with the CsPbBr_3_ QD layer exhibited the best stability, maintaining 85% of their initial PCE after 60 days, while devices without the CsPbBr_3_ QD layer retained around 75% in ambient condition (Figure 5e). This indicated that CsPbBr_3_ QD modification improved the overall device stability, protecting the perovskite layer effectively. The organic ligands in QDs positioned on both sides of the light-absorbing layer and at the grain boundaries, providing hydrophobic protection and reducing the sensitivity to humidity, enhanced the performance and stability of perovskite solar cells. The PCE statistics of 20 PSCs with different concentrations of the CsPbBr_3_ QD layer coating onto the ETL were conducted (Figure 5f). The results generally followed a normal distribution, with a noticeable increase in efficiency when the QD layers were introduced. The devices with 0.4 mg/mL of CsPbBr_3_ QD layer modification showed the highest efficiency, demonstrating good repeatability in experiments. Additionally, some optimization strategies for carbon-based HTL-free CsPbIBr_2_ PSCs in recent years are compiled in Table 3, emphasizing the potential to fabricate highly efficient and stable CsPbIBr_2_-based PSCs of our work.

**Figure 6:**
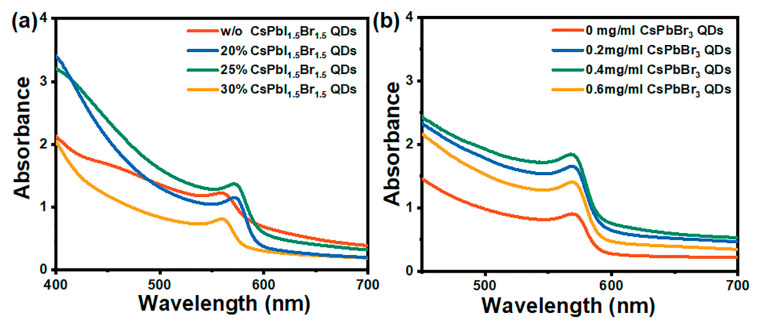
UV-vis absorption spectra of CsPbIBr_2_ films treated by CsPbI_1.5_Br_1.5_ QDs (**a**) and CsPbBr_3_ QDs (**b**).

QD treatment proved to be effective to adjust the energy band gap of a hosting material, benefiting from the quantum confinement effect dictated by the structure, composition and size of the nano-scale QDs. From the UV-vis absorption spectra (Figure 6a), when utilizing CsPbI_1.5_Br_1.5_ QDs as an additive in anti-solvent, the absorption intensity increased first and then decreased with the CsPbI_1.5_Br_1.5_ QD volume ratio increasing, and reached the strongest at 25%. At the same time, it is worth noticing that the absorption band edge presented a slight red shift via the optimized CsPbI_1.5_Br_1.5_ QDs, revealing the improvement in light harvesting. Additionally, the UV-vis spectra of the perovskite treated with CsPbBr_3_ QDs illustrated the higher absorption intensity produced by 0.4 mg/mL QDs (Figure 6b). Furthermore, deposing wider-band-gap CsPbBr_3_ QDs first onto ETL, followed by the perovskite deposition process, built a gradient energy band arrangement, which can promote charge transfer.

## 4. Discussion

Several strategies proved to be effective to improve crystallization and passivate defects, including component adjustment, interfacial engineering, additive engineering and anti-solvent engineering. On one hand, bifunctional groups, like NH_3_^+^ and COOH, were demonstrated to enhance morphological properties and passivate defects at the surface and grain boundaries simultaneously [23]. In line with the previous reports, we added CsPbI_1.5_Br_1.5_ QD additive into EA anti-solvent, a combination of optimization, in which the QD solution containing long-chain organic molecules, such as OA and OAm, can play roles as bifunctional groups, achieving higher efficiency and stability due to the passivation of the functional groups NH_3_^+^ and COOH, and the modification of hydrophobic organic molecules. Furthermore, introducing CsPbI_1.5_Br_1.5_ QDs into anti-solvent can align the energy band of perovskites and carbon electrodes to some degree owing to the quantum confinement effect. Therefore, CsPbI_1.5_Br_1.5_ QDs added into anti-solvent can not only improve the efficiency of the CsPbIBr_2_ perovskite, but also form a hydrophobic surface to protect the PSCs. On the other hand, the CsPbBr_3_ QD layer inserted between ETL and perovskite can act as nucleation seeds to assist crystal growth and decrease the trap defects. At the same time, the final device configuration of FTO/TiO_2_/CsPbBr_3_ QD/CsPbIB_2_/CsPbI_1.5_Br_1.5_ QD/carbon built a gradient energy band arrangement, which can provide better band alignment and promote carrier transportation. The mechanism of CsPbBr_3_ QD modification in the TiO_2_ ETL also involves establishing a surface enriched with both QDs and ligands on the ETL. Subsequently, the perovskite precursor solution during spin-coating initiates the deposition of Cs^+^ and Br^−^ ions on the ETL surface, serving as nuclei for perovskite growth and governing nucleation dynamics to foster comprehensive grain growth and optimize perovskite film development.

## 5. Conclusions

In conclusion, we successfully synthesized two types of QDs, namely CsPbI_1.5_Br_1.5_ and CsPbBr_3_, utilizing an advanced solvothermal method. The incorporation of CsPbI_1.5_Br_1.5_ as an additive within the anti-solvent EA can affect the crystallization kinetics of the CsPbIBr_2_ light-absorbing layers. This modification facilitated the formation of high-quality films characterized by larger crystal sizes, complete coverage, and a pinhole-free surface. Furthermore, we optimized the concentration of CsPbBr_3_ QDs, strategically incorporating them between the ETL and the perovskite layer to act as “seeds” for the crystallization of the CsPbIBr_2_ perovskite. The synergistic interplay between CsPbI_1.5_Br_1.5_ and CsPbBr_3_ QDs effectively enhanced the nucleation, crystallization, and growth processes of the CsPbIBr_2_, resulting in significantly increased grain sizes and minimized grain boundaries within the perovskite film. Due to the quantum confinement effect, the better gradient energy band alignment achieved by the two types of QD modification promoted the charge transfer. Additionally, long-chain organic molecules were located on both sides of the perovskite and at grain boundaries, forming a hydrophobic protective layer that contributed to the long-term stability of the PSCs. The resulting devices exhibited an excellent efficiency of 9.20% and demonstrated exceptional stability, retaining 85% of their initial efficiency after being stored in ambient air for 60 days. This research presents a promising methodology for the fabrication of highly efficient CsPbIBr_2_ PSCs.

## Figures and Tables

**Table 1:** Photovoltaic parameters of carbon-based HTL-free CsPbIBr_2_ PSCs fabricated by different amounts of CsPbI_1.5_Br_1.5_ QD additive in EA.

QD Ratio	Voc (V)	Jsc (mA/cm^2^)	FF (%)	PCE (%)
0%	1.12	9.39	52.11	5.48
20%	1.20	10.25	60.32	7.42
25%	1.21	10.73	61.31	7.96
30%	1.17	10.15	54.31	6.45

**Table 2:** Photovoltaic parameters of carbon-based HTL-free CsPbIBr_2_ PSCs fabricated by different concentrations of CsPbBr_3_ QD interface modification.

QDs (mg/mL)	Voc (V)	Jsc (mA/cm^2^)	FF (%)	PCE (%)
0	1.21	10.73	61.31	7.96
0.2	1.23	11.16	61.99	8.51
0.4	1.24	11.41	65.02	9.20
0.6	1.20	11.20	61.61	8.28

**Table 3:** Strategies for cabon-based HTL-free CsPbIBr_2_ PSCs in recent years.

Device Structure	PCE (%)	Long-Term Stability	Year	Ref.
FTO/TiO_2_/CsPbBr_3_ QD/CsPbIBr_2_/C	9.20	~85%@60 days in air	-	This work
FTO/c-TiO_2_/CsPbIBr_2_/C	6.50	~95%@7 days in 100 °C N_2_	2022	[42]
FTO/SnO_2_/LiF/CsPbIBr_2_/C	6.58	~72.2%@48 h in RH 60%	2022	[43]
FTO/SnO_2_/CsPbIBr_2_/CsPbIBr_2_ QDs/C	7.36	~73%@72 h in RH 70%	2023	[44]
ITO/TiO_2_/CsPbIBr_2_/C	10.15	~90%@45 days in air	2023	[45]
FTO/c-TiO_2_/CsPbIBr_2_/DES/C	7.04	~90%@200 h in RH 65–70%	2024	[46]
ITO/SnO_2_/CsPbIBr_2_/BPTAB/C	11.26	~90%@900 h in 30 °C and RH 55%	2024	[23]
FTO/c-TiO_2_/CsPbIBr_2_/C	10.67	~75%@25 days in 85 °C and RH 20%	2024	[47]
FTO/TiO_2_/CsPbIBr_2_/C	10.26	~93%@900 h in air	2024	[48]

## Data Availability

Data are contained within the article and Supplementary Materials.

## Supplementary Materials

The following supporting information can be downloaded at: https://www.mdpi.com/article/10.3390/nano14201651/s1, Figure S1: (a) SCLC curves and (b) dark J–V curves of the devices optimized by CsPbI_1.5_Br_1.5_ QDs. Figure S2: Statistics data of CsPbI_1.5_Br_1.5_ QD-optimized PSCs: (a) Voc; (b) Jsc; (c) FF; (d) PCE. Figure S3: XRD patterns of CsPbBr_3_ QD-optimized CsPbIBr_2_ films. Figure S4: SCLC curves and (b) dark J–V curves of the devices optimized by the CsPbIBr_3_ QD layer. Table S1: Statistics of the average photovoltaic parameters optimized by CsPbI_1.5_Br_1.5_ QDs.
