# Supplementary material for: Performance Enhancement of Hole Transport Layer-Free Carbon-Based CsPbIBr2 Solar Cells through the Application of Perovskite Quantum Dots
Source: Nanomaterials (Basel). 2024 Oct 14;14(20):1651. doi: 10.3390/nano14201651 (PMC11510122; doi:10.3390/nano14201651)
Supplement: Supplementary file 1 [file nanomaterials-14-01651-s001.zip › nanomaterials-3210620-supplementary.pdf]

Performance Enhancement of Hole Transport Layer-Free Carbon-Based $CsPbIBr_2$ Solar Cells through Application of Perovskite Quantum Dots

Qi Yu [1,†], Wentian Sun [2,†] and Shu Tang [2,*]

1 Huailai Shengshi New Energy Technology Co. Ltd.; Zhangjiakou 075400, China; 13811236212@139.com
2 School of Science, China University of Geosciences Beijing; Beijing 100083, China; w1063783539@163.com
* Correspondence: 3019210002@email.cugb.edu.cn
† These authors contributed equally to this work.

Characterization and Measurement

The X-ray diffraction patterns were utilized to analyze the crystalline structures of the samples. Morphological characteristics of the films were investigated using scanning electron microscopy (SEM, S-4800, Hitachi, Tokyo, Japan). UV-vis absorption spectra were measured employing a spectrophotometer (Cary 5000, Palo Alto, CA, USA), while steady-state PL spectra were examined using a photoluminescence (PL) spectrometer (Fluoro-Max, Horiba). The current density-voltage (J-V) characteristics of carbon-electrode $CsPbIBr_2$ PSCs were determined by reverse (1.3 V to 0.2V) scans in air under simulated solar illumination of 100 $mW\cdot cm^{-2}$ (AM 1.5G) employing a source meter (Keithley 2400). Additionally, incident photo-to-electron conversion efficiency (IPCE) was measured in DC mode utilizing a Zolix SCS10-X150-DZ system (Zolix, Beijing, China), while electrochemical impedance spectroscopy (EIS) was performed in the dark using an electrochemical workstation (IviumStat. H, Ivium Technologies) under a 0.5 V applied bias. To assess environmental stability, the devices underwent a 60-day duration of environmental testing in ambient atmosphere conditions, maintaining a temperature of ~25 ℃ and room humidity of 20~30%

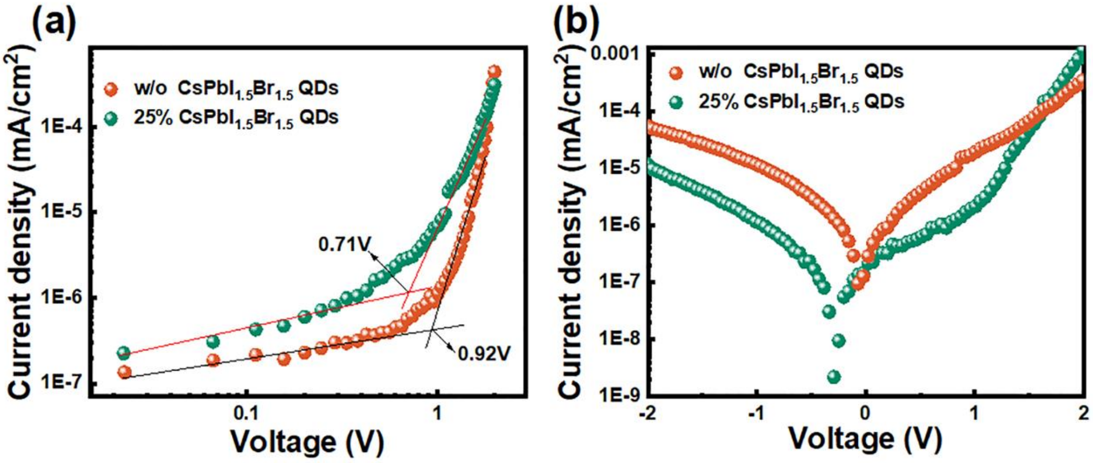

Figure S1. (a) SCLC curves and (b) Dark J-V curves of devices optimized by $CsPbI_{1.5}Br_{1.5}$ QDs

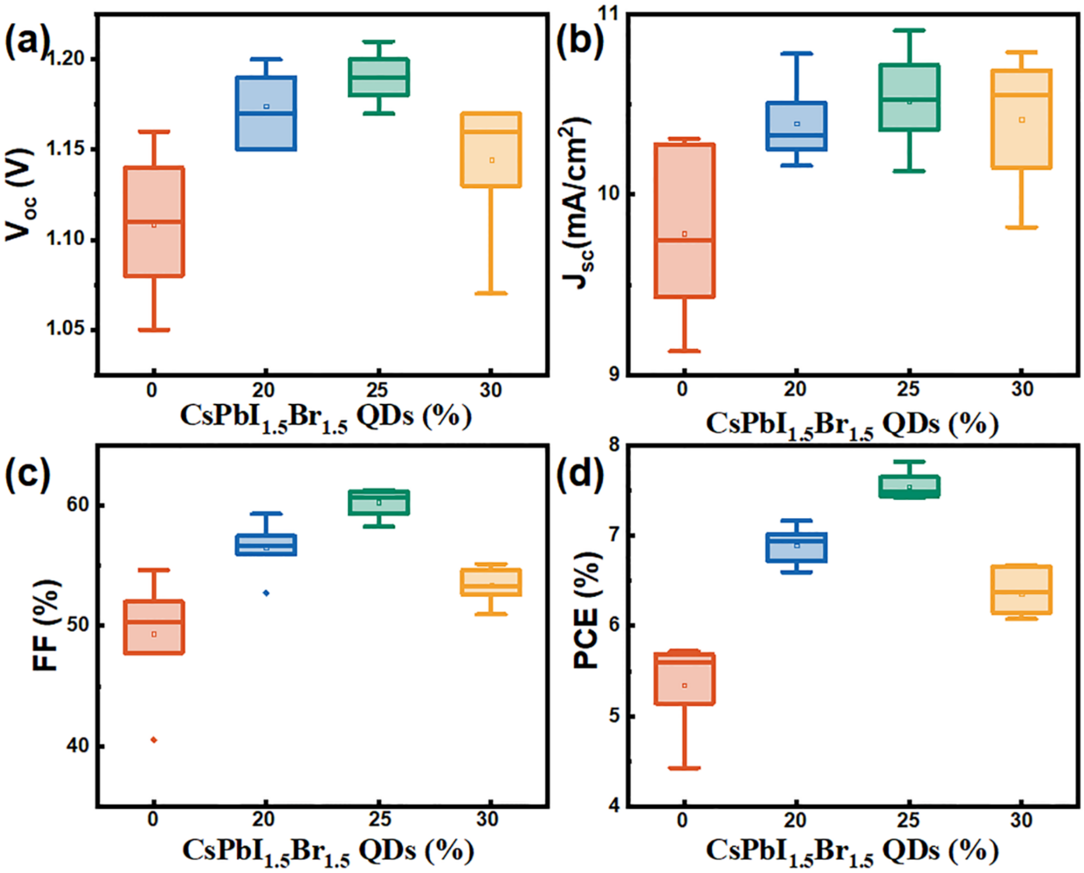

Figure S2. Statistics data of $CsPbI_{1.5}Br_{1.5}$ QDs-optimized PSCs: (a) Voc; (b) Jsc; (c) FF; (d) PCE

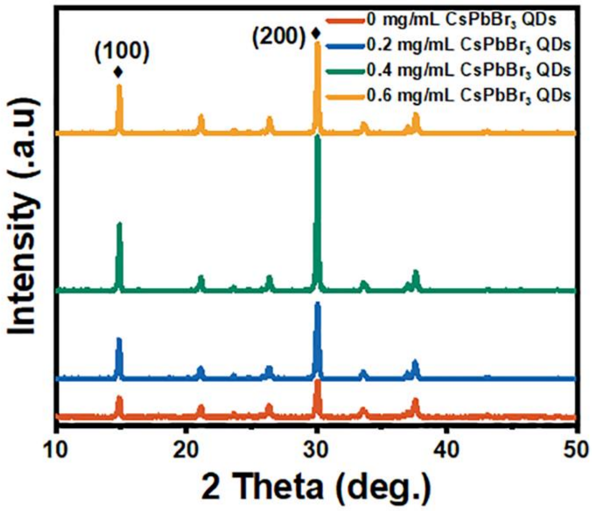

Figure S3. XRD patterns of $CsPbBr_3$ QDs-optimized $CsPbIBr_2$ films

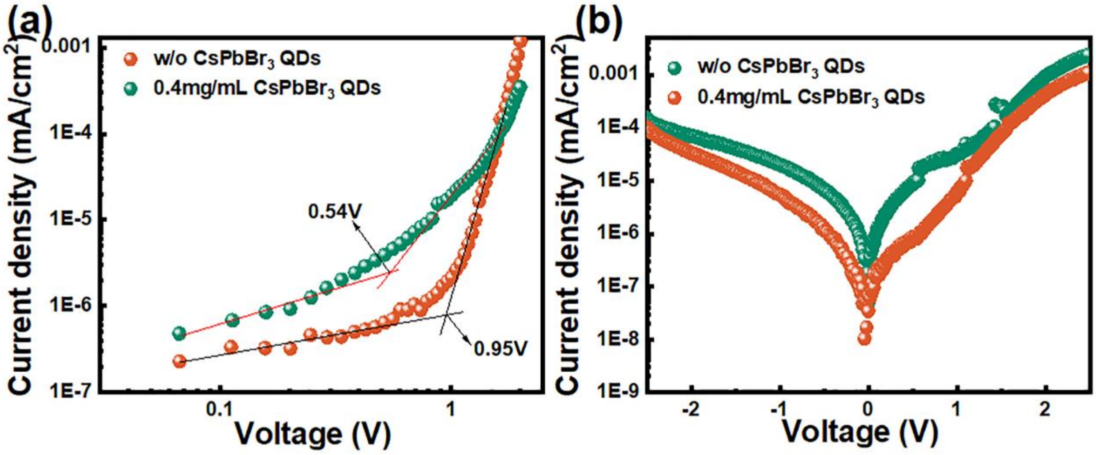

Figure S4. (a) SCLC curves and (b) Dark J-V curves of devices optimized by $CsPbBr_3$ QDs layer.

Table S1. Statistics of the average photovoltaic parameters optimized by $CsPbI_{1.5}Br_{1.5}$ QDs

QDs ratio	Voc (V)	Jsc (mA/cm^2)	FF (%)	PCE (%)
0%	1.11±0.03	9.78±0.57	49.38±4.21	5.35±0.38
20%	1.17±0.02	10.39±0.40	56.54±3.43	6.89±0.27
25%	1.19±0.01	10.52±0.42	60.31±2.17	7.55±0.28
30%	1.14±0.03	10.42±0.38	53.42±1.92	6.39±0.31
